# International Tuberculosis Conference in London

**Published:** 1921-03-12

**Authors:** 


### International Tuberculosis Conference in London.

Under the auspices of the National Association for the Prevention of Tuberculosis, the next International Conference will be held in London from Tuesday, July 26, to Thursday, July 28, inclusive. The Conference will be open to members of the International Union against Tuberculosis, and to delegates from countries within the League of Nations, and from the United States of America. The President of the International Union for the current year is Monsieur Léon Bourgeois, President of the French Senate, who will be succeeded on the occasion of the Conference in London by Professor Sir Robert Philip, of the University of Edinburgh. It is hoped that the opening address of the Conference will be given by Monsieur Léon Bourgeois. The arrangements for the Conference are under the charge of the Executive Committee of the International Union in correspondence with the Council of the National Association for the Prevention of Tuberculosis, 20 Hanover Square, W.

